# RNA sequencing profiles reveals progressively reduced spermatogenesis with progression in adult cryptorchidism

**DOI:** 10.3389/fendo.2023.1271724

**Published:** 2023-10-30

**Authors:** Weihao Sun, Xinhui Zhang, Lei Wang, Guanyu Ren, Shuguang Piao, Chenghua Yang, Zhiyong Liu

**Affiliations:** ^1^ Department of Urology, Changhai Hospital, Second Military Medical University, Shanghai, China; ^2^ Shanghai Key Laboratory of Cell Engineering, Shanghai, China

**Keywords:** cryptorchidism, infertility, RNA sequencing, spermatogenesis, testis

## Abstract

**Introduction:**

The fertility of cryptorchidism patients who didn’t perform corrective surgery will decrease with age. Herein, we elucidate the histological alterations and underlying molecular mechanism in patients with an increase in the disease duration from 20 to 40 years.

**Methods:**

Testicular tissues were obtained from three patients with cryptorchidism, ranging in age from 22 to 44 years. Three benign paracancerous testicular samples of matched ages were used as controls. The normal and undescended testicular tissues were stained with hematoxylin and eosin (HE) and immunofluorescence and all six testicular samples were subjected to RNA sequencing. RNA sequencing data were subjected to gene set enrichment analysis (GSEA), Kyoto Encyclopedia of Genes and Genomes (KEGG), protein-protein interaction (PPI) network analysis, and Gene Ontology (GO) searches. Real-time reverse transcriptase polymerase chain reaction was used to confirm the DEGs.

**Results:**

The seminiferous tubules’ basement membrane thickens with age in healthy testes. As the period of cryptorchidism in the cryptorchid testis extended, the seminiferous tubules significantly atrophy, the number of spermatogenic cells declines, and the amount of interstitial fibrous tissue increases in comparison to normal tissues. The number of germ cells per cross-section of seminiferous tubules was significantly lower in cryptorchidism than in normal testicular tissues, according to immunofluorescence staining, but the number of Sertoli cells remained stable. RNA sequencing analysis identified 1150 differentially expressed genes (DEGs) between cryptorchidism and normal testicular tissues (fold change >2 and p<0.05), of which 61 genes were noticeably upregulated and 1089 were significantly downregulated. These genes were predominantly linked to sperm development and differentiation, and fertilization, according to GO analysis. Meiosis pathways were significantly downregulated in cryptorchidism, according to KEGG pathway analysis and GSEA (P<0.001). PPI analysis was used to identify the top seven downregulated hub genes (*PLCZ1, AKAP4, IZUMO1, SPAG6, CAPZA3*, and *ROPN1L*), which were then further verified by qPCR.

**Discussion:**

By describing the histological changes and differential gene expression patterns in adult cryptorchid patients of different age groups, we discovered the progression mechanisms of undescended testes in adults with aging and identified seven significantly downregulated hub genes (*PLCZ1, AKAP4, IZUMO1, SPAG6, CAPZA3*, and *ROPN1L*) in cryptorchid testis compared to normal testicular tissues. These genes played a role in the process of spermgenesis and are directly linked to the steady decline in fertility caused by cryptorchidism. Our study provided a better understanding of the molecular mechanisms underlying the loss of spermatogenesis in adult cryptorchidism, and give support for the development of adult cryptorchidism treatments.

## Introduction

Cryptorchidism (also known as ndescended testis (UDT)) refers to the failure of one or both testes to migrate through the inguinal canal and into the scrotum due to the abnormal developmental process during fetal life. Cryptorchidism is one of the most common congenital anomalies in male infants, with an incidence rate of 1.6-9% varying across different countries (1). Cryptorchidism is associated with high rates of male infertility, as nearly 10% of males with fertility issues have a history of cryptorchidism and orchidopexy, and 20–27% of male adults affected by azoospermia and 3–8% affected by oligo-terato-asthenospermia were previously diagnosed with testicular maldescent (2). For congenital cryptorchidism, it is recommended to perform corrective surgery within the first 18 months of birth (3, 4).

Therefore, there is a decreasing trend in the average age of patients undergoing UDT surgery. However, studies showed that even if surgery is performed before 6 months of age, 31% of UDT patients still will experience a decrease in sperm count in adulthood (5). In addition to impaired fertility, the risk of malignant tumors has been shown to increase with age at which testicular fixation surgery is performed (6–9). Therefore, postponing surgery for patients with cryptorchidism may have a number of negative consequences. After puberty, patients with undescended testes are now uncommon due to the widespread advice for early orchiopexy within the first year of life. As a results, it is unclear what the progressive process is if the testis is preserved in the undescended state in adulthood. In order to develop appropriate treatments for chronic cryptorchidism, it is important to fully understand the mechanisms underlying persistent cryptorchidism.

It has been reported that there is a significant reduction in the number of germ cells, and relative increase in the percentage of sertoli cells in patients with cryptorchidism. Testes that remain undescended are associated with progressive loss of germ and Leydig cells (10–12). If cryptorchidism is a progressive disease, the longer the testis remained in the undescended environment, the lesser the number of germ cells and/or Leydig cells (13). However, due to the limited cases that patients take the orchiopexy after adulthood, few studies were carried out to investigate how the cryptorchidism actually progress as the testis remain undescended for longer periods. Moreover, most studies on prolonged cryptorchidism were based on animal models (11, 14–16), which cannot simulate the real human cryptorchidism development and progression. Here, we collected testicular tissue samples from three cryptorchidism patients who underwent orchidopexy at the ages from 22 to 44. Three para-cancerous normal testis tissues were also collected from testicular cancer patients as control with matched ages. By describing the histopathological changes and differential gene expression in each age group, we provided an overview of the development process of adult cryptorchidism with different undescended testis time, and identified the molecular mechanisms that lead to fertility impairment in adult UDT patients, thus provided a research foundation for the future restoration of fertility in adult UDT patients.

## Materials and methods

### Recruitment of clinical patients

**Figure 1:**
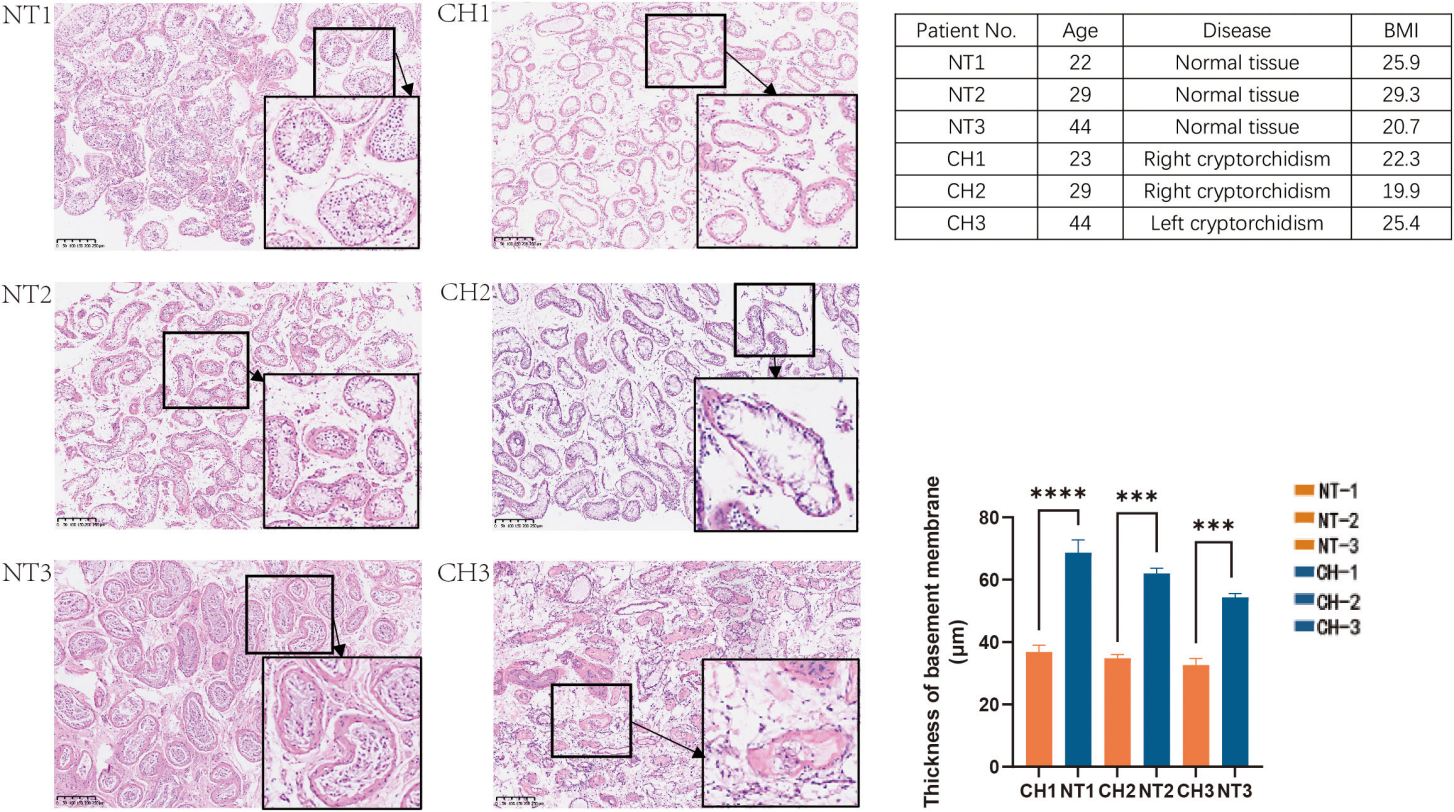
HE staining of the normal and undescended testis in different age groups. NT is for normal testis and CH is for cryptorchidism. Each image’s number corresponds to the patient information in the table on the right. (H&E stain, x40, Bar, 250 mm). Data are mean ± SD of three independent experiments. ***P < 0.001, ****P < 0.0001, ANOVA.

With Changhai Hospital’s ethical approval (CHEC2021-086), testicular tissues were obtained from three cryptorchid patients and three testicular cancer patients who underwent orchidopexy. The undescended testis tissues from the cryptorchid patients and the para-cancerous benign testis tissues from testicular cancer patients were collected. Clinical data of cryptorchid patients and testicular cancer patients are shown in **Figure 1**. The collected tissues were divided into 2 portions, 1 was stored in 4% paraformaldehyde (PFA) immediately, and another 1 was fast frozen with liquid nitrogen and then stored at -80°C until use.

The inclusion criteria for cryptorchidism patients were: (1) Patients who have confirmed cryptorchidism by ultrasound; (2) Patients without reproductive history; (3) Patients aged between 18 and 50 years. The inclusion criteria for testicular cancer patients were: (1) Patients who have histologically testicular cancer with operations; (2) Patients with reproductive history; (3) Patients without hormonal treatment history (4) Patients aged between 18 and 50.

Exclusion criteria were as follows: (1) Patients with severe diseases including liver, kidney, hematopoietic, and cardiovascular; (2) Patients with testicular injuries; (3) Patients who had received medication within 6 months.

### Histological examination

Testicular tissues were fixed in 4% PFA for 24h, embedded in paraffin, and sliced into 6 μm thickness. The sections were stained with Hematoxylin and eosin (H&E) and observed under a microscope.

### Immunofluorescence staining

Immunofluorescence staining was performed based on previously established protocol (17). Briefly, slides were first stained with a rabbit anti-*DAZL* antibody (Cat. No. ab215718, Abcam; 1:250); a rabbit anti-*DDX4* antibody (Cat. No. ab270534, Abcam; 1:250); a rabbit anti-SOX9 antibody (Cat. No.AB5335, Millipore;1:250); a rabbit anti-*SYCP3* antibody (Cat. No. ab15093, Abcam;1:250); or a rabbit anti-*STAR* antibody (Cat. No. 12225-1-AP, Proteintech;1:200). After rinsing, tissues were stained by secondary antibodies (a donkey anti-rabbit Alexa 647 (Cat. No.ab150075, Abcam; 1:1000)) at room temperature for 2 h. Nuclei were counterstained by the mounting medium with DAPI (Cat. No. ab285390, Abcam). Images were taken by a confocal microscope (Leica SP-8, Leica Corporation, German).

### Whole transcriptome sequencing

A total of 2 μg RNA per sample was used as input material for the RNA sample preparations. RNA-seq library preparation was performed by Novogene (Novogene Corporation Inc., Beijing, China). According to the manufacturer’s instructions, total RNA was isolated from tissues with the Qiagen RNeasy Plus Mini Kit (Qiagen). Then RNA quality was determined by 2100 Bioanalyser (Agilent, CA, USA) and quantified using the ND-2000 (NanoDrop Technologies, CA, USA). Only high-quality RNA sample (OD260/280 = 1.8~2.2, OD260/230≥2.0, RIN≥6.5, 28S:18S≥1.0, >1μg) was used to construct sequencing library. RNA-seq transcriptome library was prepared following TruSeqTM RNA sample preparation Kit from Illumina (San Diego, CA, USA) using 1μg of total RNA. Shortly, messenger RNA was first isolated according to polyA selection method by oligo(dT) beads and then fragmented by fragmentation buffer. Secondly double-stranded cDNA was synthesized using a SuperScript double-stranded cDNA synthesis kit (Invitrogen, CA, USA) with random hexamer primers (Illumina). Library quality was assessed on the Agilent Bioanalyzer 2100 system.

### RNA-seq analysis

The RNA sequencing data obtained were normalized using the Limma Bioconductor package in the R computing environment. After normalization and batch effect removal, the fluorescence intensities were transformed into a log2 base prior to further analysis. Differential gene expression levels were determined by comparing processed array signals in different samples. Genes with an FDR of <0.05 were considered statistically significant. Pathway and gene ontology analyses were performed using the Ingenuity IPA program for DEGs. PPI networks were established by Search Tool for the Retrieval of Interacting Genes Database (STRING) based on the combined score > 0.9 and visualized by Cytoscape 3.9.1 (version 4.2.2) to reveal the interactions among proteins of common DEGs.

### RNA extraction and quantitative real-time PCR array

Trizol (Cat. No. R0016, Beyotime, Beijing, China) was used to extract RNA from tissue samples according to the manufacturer’s instructions. Use NanoDrop ND-1000 to determine the concentration of RNA, and store the extracted RNA in a refrigerator at -80°C. Using 500 ng total RNA as a template, cDNA was synthesized with cDNA Synthesis Kit (Cat. No. R312, Vazyme, Beijing, China). Samples were stored at -20°C and subjected to qPCR using a StepOnePlus Real-Time PCR System (Applied Biosystems). Each qPCR sample was performed in a triplicate and 10 μL reaction containing 2x SYBR Green qPCR Master Mix (Cat. No. R711-02, Vazyme, Beijing, China), 10 nM forward and reverse primers, and 2 μL cDNA. The qPCR protocol was executed for 45 cycles and each cycle consisted of denaturation at 95°C for 15 s, annealing at 60°C for 1 min, and extension at 72°C for 1 min. Using β-actin as internal controls, quantitative PCR analysis was performed to quantify the relative mRNA expression of targeted genes. Define the result of qPCR from the threshold cycle (Ct) and use the 2-△△Ct method to calculate the relative expression level. The primer pairs specific for various genes used in our experiments are listed in **Table 1**.

**Table 1:** Bases sequences of primers.

Primer	Forward (5′-3′)	Reverse (5′-3′)
SPAG6	AAGAGCCAAAGCAGCAGTCT	AATGGACAGGCAGGTGTAATG
CAPZA3	GCCACCAACACTGCCAAAAA	AGTCGCCCATTACATTGTGGT
AKAP4	GCGTACTCTGATACTACAATGATGT	CAGGGTGGACACATCGACAA
AKAP3	CCCAGGACTGGAAAATGGACA	TTGGACGTTTCCCCACCAAA
PLCZ1	CTCTACCATCACCAGAGGCAC	ACCCCTGTTTCCTTGTCTTGA
IZUMO1	GCTCTCGATTTCACGCAACC	GCAGTGTGGCCTCATGCTAA
SPAG6	AAGAGCCAAAGCAGCAGTCT	AATGGACAGGCAGGTGTAATG
ROPN1L	CGCGGGCTATTTTTCAGCTC	GCTTGTGGTGACACTGCTTG
ACTB	ACAGAGCCTCGCCTTTGC	GATATCATCATCCATGGTGAGCTGG

### Statistical analysis

All experiments were repeated at least three times. Data were presented as mean ± standard deviation. Two-way ANOVA was used for comparison between two groups. Test standard α=0.05, p<0.05 was considered statistically significant, p<0.01 was very significant, and p<0.001 was extremely significant. Statistical analysis were performed using GraphPad Prism (GraphPad, CA, USA).

## Results

### Histological differences between cryptorchidism and normal testis

First, we looked at the histological traits of patients with cryptorchid testicular tissues. Para-cancer benign tissues that were matched by age were used as controls. According to histological analysis, the seminiferous tubules’ basement membrane thickens with age in normal testes (**Figure 1**). In contrast, the cryptorchid testes’ seminiferous tubules markedly atrophied, the amount of spermatogenic cells decreases, and the quantity of interstitial fibrous tissue grows in comparison to normal tissues as the duration of cryptorchidism increased. All of these histological characteristics of cryptorchid individuals showed that, in comparison to normal testicles, cryptorchid testes were extensively injured. (**Figure 1**).

**Figure 2:**
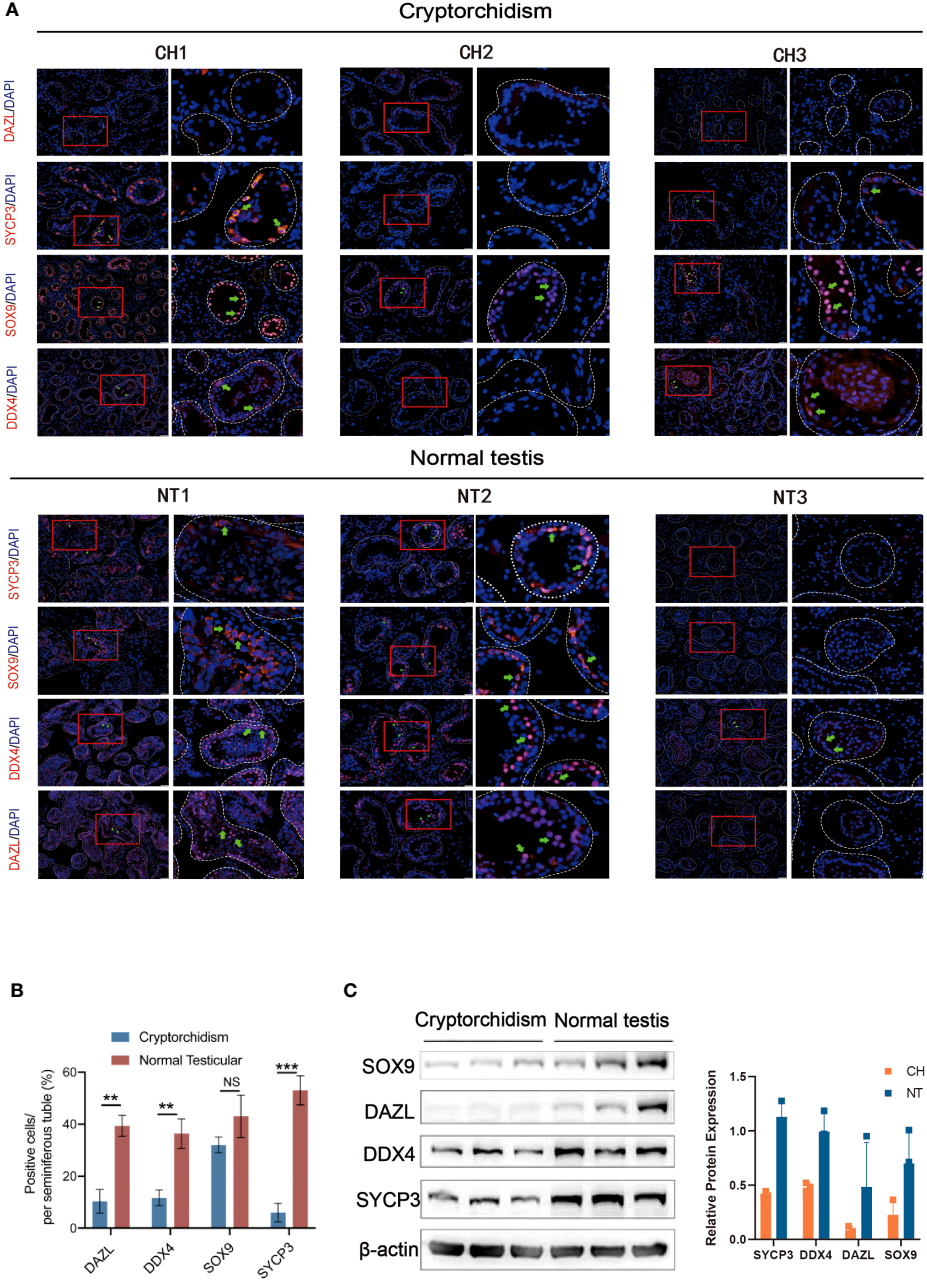
The expression of DAZL, DDX4, SOX9 and SYCP3 between cryptorchidism and normal testis. **(A)** IF staining of DAZL, DDX4, SOX9, and SYCP3; **(B)** Quantification of the number of DAZL, DDX4, SOX9 and SYCP3 positive cells per seminiferous tubule. **(C)** Western blot of the expression of SOX9, DAZL, DDX4 and SYCP3. Data are mean ± SD of three independent experiments. **P<0.01, ***P < 0.001, ns P>0.05, ANOVA.

Next, we performed immunofluorescence staining to evaluate the composition of different cellular content within the testis, including germ cells, Sertoli cells, and spermatid during testicular development in normal and cryptorchid testis (**Figure 2A**). Deleted in Azoospermia-like (*DAZL*, which is located in the nucleus of spermatogonia but relocated in the cytoplasm during meiosis where it persists in spermatids and germ cells), DEAD-Box Helicase 4 (*DDX4*, specifically expressed in the germ cell lineage), SRY-Box Transcription Factor 9 (*SOX9*, marker of Sertoli cells), and Synaptonemal Complex Protein 3 (*SYCP3*, relevant with meiosis) proteins were stained in testicular tissues to evaluate cellular composition changes involving in spermatogenesis between cryptorchidism and normal testis. We found that cryptorchid testis had lower *DAZL, DDX4*, and *SYCP3* positive cells in seminiferous tubule compared with normal testis, consistent with previous observations by other investigators. A small number of germ cells still exist in cryptorchid patients of the 20-year-old group. However, as time progresses, the number of germ cells in cryptorchid patients continues to decrease, indicating that the damage to fertility caused by cryptorchidism accumulates over time. No significant difference in *SOX9*-positive cells were observed between cryptorchid and normal testis, suggesting that cryptorchidism markedly inhibited spermatogenesis and induced the arrest of spermatogonia without apparently affecting the Sertoli cell. Similar results were observed by examining the expression of *SOX9, DAZL, DDX4* and *SYCP3* in the testis tissues of both groups by western blot (**Figure 2B**).

### Differential expression of genes between the cryptorchidism and normal testis

**Figure 3:**
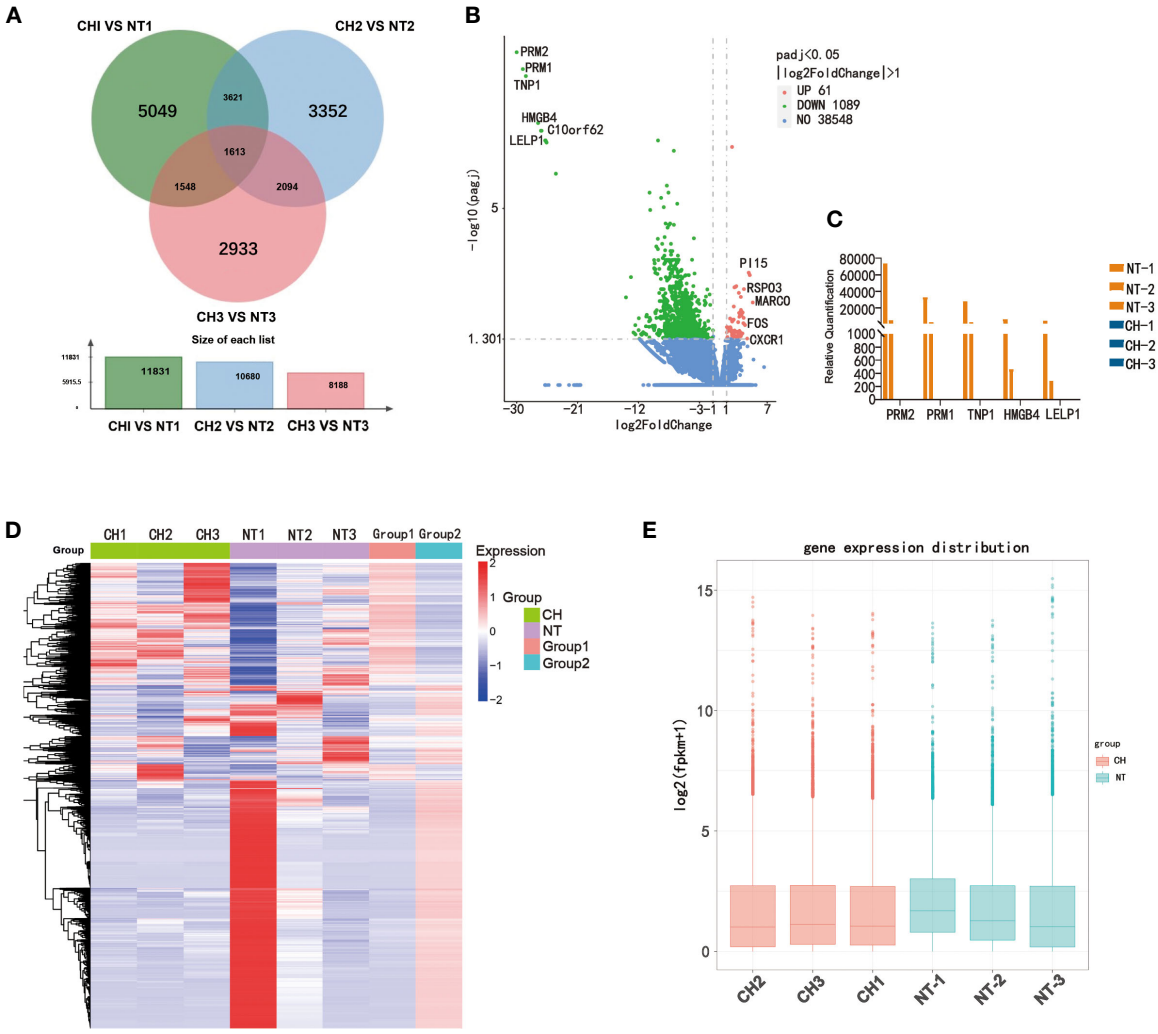
Differentially expressed genes between normal testis and cryptorchid testis. **(A)** A Venn diagram showed the intersection of genes acquired from the CH1 *VS* NT1 group (green), CH2 *VS* NT2 group (blue) and CH3 *VS* NT3 group (red). **(B)** The volcano map showed DEGs between cryptorchidism and normal testis. The green and red dots are the downregulated genes and the upregulated genes, respectively with statistical significance (log2 [Fold change] >2 and p < 0.05). **(C)** The expression of PRM2, PRM1, TNP1, HMGB4 and LELP1 in different samples. **(D)** Heat map shows the relative expression of DEGs between cryptorchidism and normal testis **(E)** Boxplot, a method of describing data in terms of minimum, first quartile (25%), median (50%), third quartile (75%), and maximum. The abscissa is the sample name, and the ordinate is the signal value of the probe after log2.

To elucidate the mechanisms by which cryptorchidism impairs fertility with age increasing, we performed RNA sequencing of cryptorchid and normal testis. We first analyzed the differences in expression levels between cryptorchid and normal testis using venn diagram and volcano plot (**Figures 3A, B**). The Venn diagram reveals significant differences in the detected genes among different age groups, with only 1613 common genes (**Figure 3A**). The differential genes between cryptorchid testis and normal testis were screened by fold change and p-value statistical criteria. The top 5 significantly downregulated genes in cryptorchid testis were *PRM2, PRM1, TNP1, HMGB4* and *LELP1*, which also showed a decreasing trend with age in normal testicular tissues, indicating their significant role in male reproduction (**Figure 3C**). Additionally, the heatmap showed significant differences in gene expression between the two groups (**Figure 3D**). A box plot was used for visualizing the intensities of expression values of genes in two different groups after normalization, which also showed a significant decrease in gene expression levels in the cryptorchidism group (**Figure 3E**). These results indicate significant differences in gene expression patterns between cryptorchid and normal testicular tissues, and the expression levels of these DEGs are moderately correlated with age. It suggests that these DEGs may play important roles in male fertility.

### Bioinformatics analysis of differentially expressed genes

**Figure 4:**
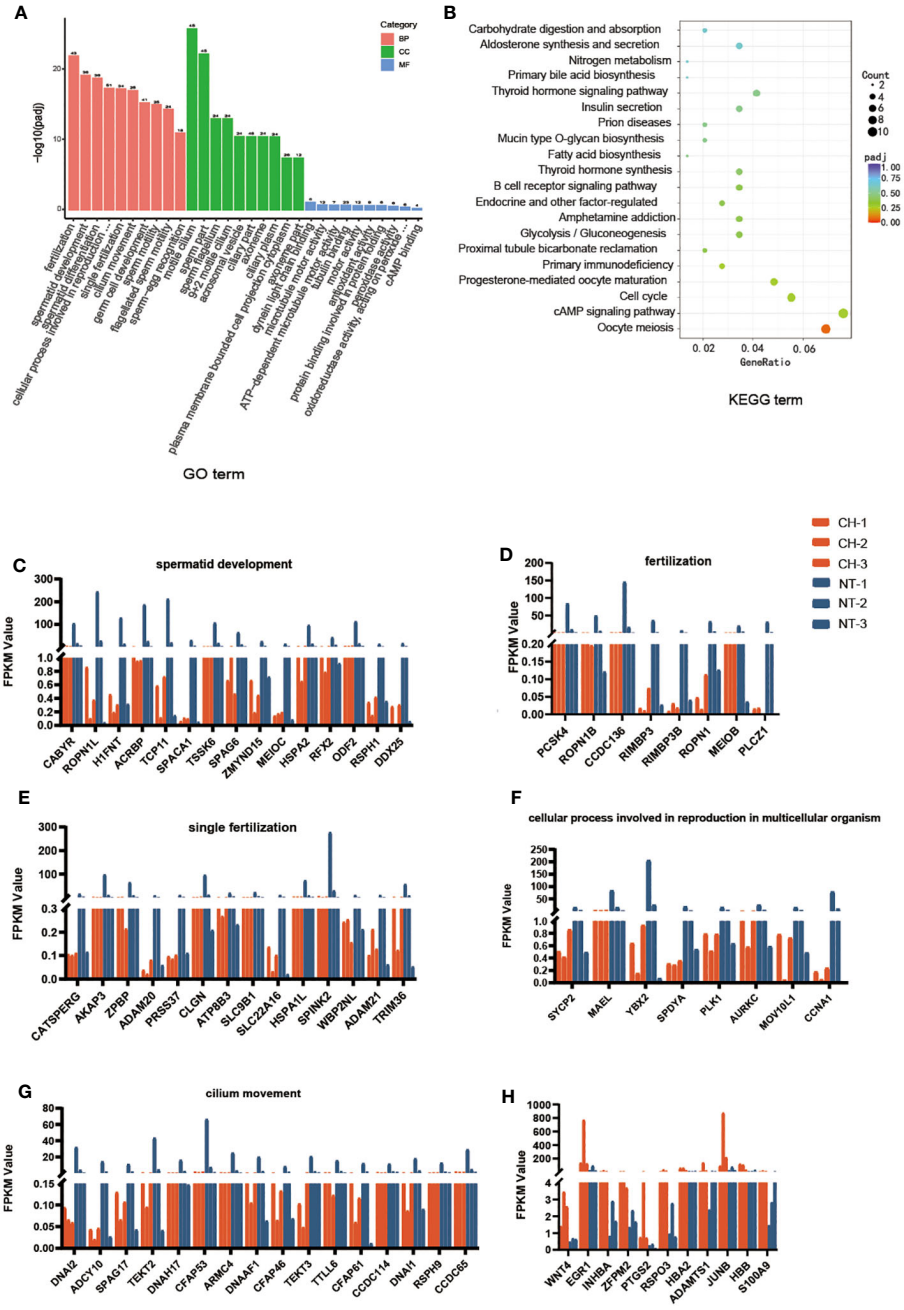
Biological chracteristics of the differentially expressed genes. **(A)** GO enrichment annotations of DEGs between cryptorchidism and normal testis. The orange bar is the Biological Process, the green bar is the Cellular component and the blue bar is the Molecular function. **(B)** KEGG pathway enrichment analysis revealing key signaling pathways of DEGs. **(C–H)** The expression of genes within the first six upregulated and downregulated GO biological processes in different samples.

GO and KEGG analyses were carried out to elucidate the function of the differently expressed genes. According to GO analysis, DEGs play a major role in fertilization, spermatid development and differentiation, among other processes (**Figure 4A**). According to KEGG pathway analysis, the disrupted genes were prominent in cell cycle, meiosis, and cAMP signaling (**Figure 4B**). We next looked at the expression of the genes connected to the top six GO biological processes. The expression of these genes was shown to be lowered in cryptorchidism, and the decreased level became more noticeable with age (**Figures 4C–H**). These genes are mostly involved in sperm motility and germ cell development, which provides underlying molecular mechanism explanations for the histological characteristics of cryptorchidism as shown in **Figure 1**.

**Figure 5:**
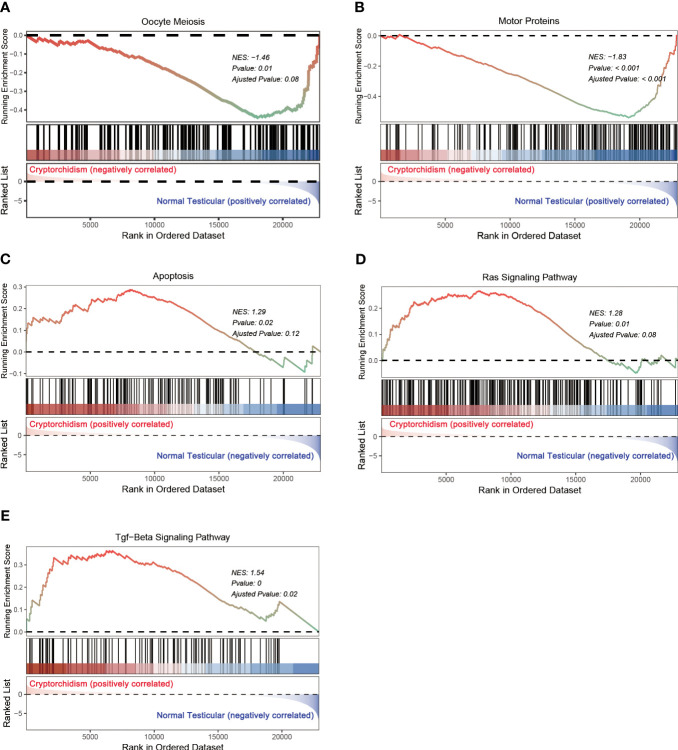
GSEA analysis of differential expression genes. **(A–E)** represents the signaling pathway enriched in the DEGs.

Gene set enrichment analysis (GESA) was also performed to further explore the impacts of alternated genes on biological pathways. The top altered gene sets were shown in **Table 2**. In addition to oocyte meiosis pathway as identified in GO analysis, motor proteins, apoptosis, Ras signaling pathway, and TGF-β signaling pathway were the top enriched gene sets in cryptorchid testis. oocyte meiosis and motor proteins pathways were down regulated in cryptorchid testis, while apoptosis, Ras signaling pathway, and TGF-β signaling pathway were upregulated in cryptorchid testis (**Figure 5**). However, “cAMP signaling pathway” did not show significant differences in GSEA analysis. Research has shown that motor proteins are expressed at high levels in the testes and play an important role in telomere movement during meiotic division (18). The misplaced position caused by cryptorchidism can lead to thermal stress injury of the testes, activating the apoptotic pathway and resulting in impaired testicular and sperm quality (19). Ras signaling pathway has been studied extensively in the biological context since it regulates the cell cycle and cellular growth, differentiation, metabolism, and senescence. Thus, it appears that Ras signaling is essential for the normal development of mammalian tissues. Therefore, it is not surprising that the dysregulation of this pathway can have profound consequences on testis tissue development of cryptorchidism (20). A previous study has shown that exposure to the antiandrogen flutamide *in utero* can lead to failure of testicular descent in adult rats, and changes in the molecular components of the TGF-β signaling system have been identified (21). The results indicate that cryptorchidism leads to alterations in various reproductive pathways, resulting in apoptosis or differentiation blockade of germ cells, ultimately impairing fertility.

**Table 2:** Gene sets enriched in phenotype high.

Gene set name	NES	NOM p-value	FDR q-value
Oocyte meiosis	-1.45567	0.011286	0.064119
Motor proteins	-1.83254	4.15E-07	0.000114
Apoptosis	1.291074	0.019275	0.095915
Ras signaling pathway	1.275003	0.01148	0.064119
TGF-beta signaling pathway	1.53573	0.001258	0.014968

NES, normalized enrichment score; NOM, nominal; FDR, false discovery rate.

Gene sets with NOM p-value <0.05 and FDR q-value <0.25 were considered as significant.

**Figure 6:**
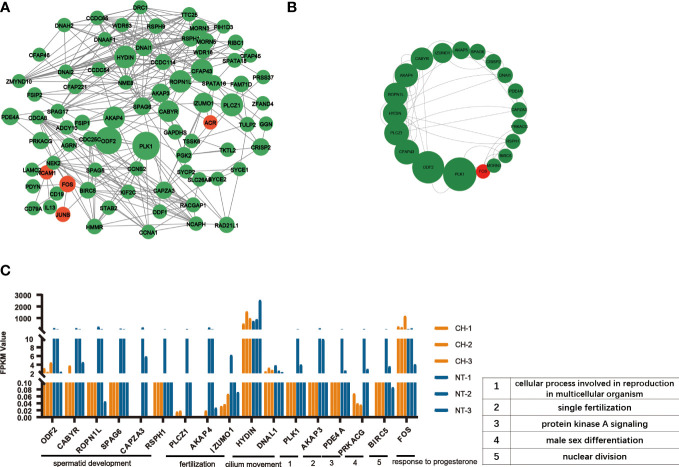
PPI network analysis of DEGs. **(A)** Protein-protein interaction network of DEGs. Each node represented a protein. The width of the edges was proportional to the score of protein-protein interaction. **(B)** The top 20 densely-connected modules identified by Betweenness. Red nodes were upregulated ones, green downregulated ones. **(C)** The expression of top 20 hub gens in different samples. The biological processes associated with these hub genes were noted.

Moreover, the STRING online database was used to analyze the protein-protein interactions among the DEGs. The results were extracted and visualized using Cytoscape software. After excluding the isolated nodes, we chose the top 80 hub genes based on degree method scores in the PPI network, as is shown in **Figure 6A**. Then we selected top 20 hub genes with the highest degree (the number of direct connections that a node has with other nodes) to investigate their effect on the development of cryptorchidism (**Figure 6B**).Then we found the GO function of these genes are very similar with the first six GO biological process (**Figure 6C**). So we think these genes may play key roles in the infertility of cryptorchidism with increase in age.

### qPCR validation for gene expression

**Figure 7:**
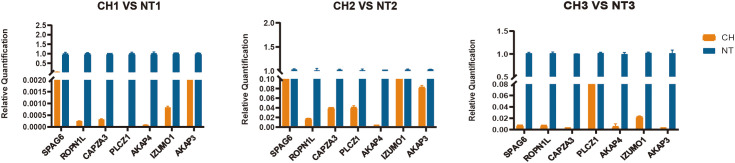
Validation of selected top 7 genes by quantitative RT-PCR. The relative expression levels of 7 genes were determined by real-time RT-PCR. All values represent mean and standard deviation. The orange bar is the cryptorchidism and the blue bar is the normal testis.

According to the extent of difference between cryptorchidism and normal testis, the expression of seven downregulated genes (*PLCZ1, AKAP4, AKAP3, IZUMO1, SPAG6, CAPZA3*, and *ROPN1L*) were further selected for validation by qPCR (**Figure 7**). We confirmed that these genes were significantly downregulated in the cryptorchid testis, which is consistent with the RNA sequencing results.

*PLCZ1* encoding 1-phosphatidylinositol 4,5-bisphosphate phosphodiesterase zeta-1 (*PLCζ*) which is the primary stimulus for egg activation and early embryonic development. If the expression levels of sperm plcζ was reduced, it will lead to male infertility (22).

*AKAP4* (A-kinase anchoring protein 4) and *AKAP3* (A-kinase anchoring protein 3) genes are structural proteins of the fibrous sheath which play important roles in sperm formation and motility (23). *AKAP4* is mainly responsible for regulating the signal transduction and metabolic pathways that support sperm motility and capacitation. Oxidative stress will destroy the *AKAP4* which results in the defective sperm function associated with male infertility (24).

*IZUMO1* is the only cell surface protein expressed on sperm that is known to be essential for sperm–egg interaction *in vivo* which plays an essential role in recognition or adhesion between the sperm and egg. When sperm and egg bind, the *IZUMO1* protein binds to the JUNO protein on the egg membrane, allowing the sperm to penetrate the egg membrane and enter the egg, thereby initiating fertilization (25).

*Spag6* (sperm-associated antigen 6) is essential for sperm flagellar motility which it is important for the maintenance of the structural integrity of mature sperm. Research indicate that if *Spag6* is deficient, their sperm was marked motility defects and was morphologically abnormal with frequent loss of the sperm head and disorganization of flagellar structures, including loss of the central pair of microtubules and disorganization of the outer dense fibers and fibrous sheath (26).

*CAPZA3* (Capping Protein Muscle Z-line Alpha 3) localized to the anterior head in intact sperm similar to *IZUMO1* (27). *CAPZA3* plays a role in maintaining polymerized actin during spermiogenesis (28).

*ROPN1L* (Ropporin 1-like) gene encodes a sperm head-specific protein that plays an important role in sperm development and maturation. Sperm proteins *ROPN1* and *ROPN1L* bind *AKAP3*. The mice deficient in *ROPN1L* (*RLKO*) will reduce sperm motility (29).

Expression of candidate genes was validated by qPCR based on the previously frozen tissue samples. Consequently, the result of RT-qPCR (**Figure 7**) has a similar expression to the result of RNA sequence. Taking together, these results shed light on potential genes that might be relevant to comprehend human fertility disorders caused by cryptorchidism.

## Discussion

Testes descending into the suitable position of the scrotum is a critical process for the development of reproductive system. Failed testes descending will lead to cryptorchidism which is a high risk factor of fertility. Now newborns who has cryptorchidism usually receive the corrective surgery before 1 year old. However, there are a small portion of patients did not receive the correction surgery even after adulthood due to various reasons. Additionally, as the undescended testis persists longer in the abdomen, the tissue structure of cryptorchidism deteriorates, which causes fertility to gradually drop. For instance, those who did not have corrective surgery, as depicted in **Figure 1**, exhibited aberrant and atrophic seminiferous tubule shape with missing germinal cells, and the effects of damage progressively aggravated. Additionally, immunofluorescence labeling reveals that the majority of cell populations in adult cryptorchidism decreased with age in comparison to normal testis (**Figure 2**). However, currently there is a lack of research on adult patients with cryptorchidism who wish to preserve their fertility and avoid malignant transformation.

Hence, identifying the underlying mechanism of progressive cryptorchidism would be useful for early diagnosis and treatment. In this study, we performed RNA sequencing in undescended testes from adult human cryptorchidism aiming to find the key genes involved in the progression of cryptorchidism. We identified 1150 DEGs in undescended testes in comparison to age-matched normal testis, including 61 upregulated and 1089 downregulated genes. Most of the DEGs are downregulated in cryptorchidism, indicating that incorrect positioning leads to the inability of many genes to be expressed in the testes. GO analysis showed that these DEGs are mainly enriched in processes such as fertilization, spermatid development, spermatid differentiation, single fertilization, and cilium movement. These biological processes are closely related to testicular development, spermatogenesis, and germ cell differentiation. Furthermore, KEGG analysis also showed that the oocyte meiosis signaling pathway, cAMP signaling pathway, and cell cycle signaling pathway were affected. GSEA analysis further confirmed that the oocyte meiosis signaling pathway was significantly downregulated in adult cryptorchidism patients, which is essential for spermatogenesis and fertility (30). Furthermore, we found that the histological features and gene expression patterns also change with aging, providing an overview for the aging process in the testis tissues. Additionally, we used PPI analysis to identify top 20 hub genes inside these DEGs (**Table 3**). The majority of these genes were involved in spermatid development, fertilization, cilium movement, etc., which is similar with the findings from GO, KEGG, and GESA study. In the end, we chose 7 genes and used q-PCR to confirm that their expression was consistent with the findings of RNA-seq.

**Table 3:** Top 20 hub genes with higher connection with others.

Number	Gene name	Degree
1	PLK1	8926.88
2	ODF2	8402.89
3	CFAP43	5667.07
4	PLCZ1	5665.8
5	HYDIN	5467.25
6	ROPN1L	5303.34
7	AKAP4	5187.02
8	CABYR	4770.95
9	IZUMO1	3818
10	AKAP3	2961.71
11	SPAG6	2335.78
12	CRISP2	2234
13	DNAI1	2194.98
14	PDE4A	2166.01
15	CAPZA3	2148.74
16	PRKACG	2096.11
17	RSPH1	2007.12
18	BIRC5	1727.41
19	MORN3	1704
20	FOS	1674.12

Degree was calculated using CytoNCA which representing the correlation of genes.

In conclusion, our study significantly increased our understanding of how adulthood cryptorchidism develops and had some implications for treating adulthood cryptorchidism by providing an overview of the transcriptomic profiles in the development of adulthood cryptorchidism and identifying key genes and associated pathways involved in its progression. Our research also offered hints about changes in spermatogenesis with age. Understanding the functions of the major genes played in the development of cryptorchidism proved to require further research.

## Conclusions

In this study, we investigated the histological changes and identified 1150 DEGs in adult cryptorchid patients of different age groups, which are mostly associated with spermatid development and fertilization pathways. We selected seven significantly downregulated genes (*PLCZ1, AKAP4, IZUMO1, SPAG6, CAPZA3*, and *ROPN1L*) in cryptorchid testis, and validated their expression by q-PCR. Our research findings contribute to a better understanding of molecular mechanism associated with the failure of spermatogenesis in adult cryptorchidism and provide evidence for treatment development for adult cryptorchidism.

## Data availability statement

The datasets presented in this study can be found in online repositories. The names of the repository/repositories and accession number(s) can be found in the article/supplementary material.

## Ethics statement

The studies involving humans were approved by Changhai Hospital’s ethical approval (CHEC2021-086). The studies were conducted in accordance with the local legislation and institutional requirements. The participants provided their written informed consent to participate in this study.

## Author contributions

WS: Resources, Writing – original draft, Writing – review & editing. XZ: Methodology, Supervision, Writing – original draft, Writing – review & editing. LW: Conceptualization, Formal Analysis, Investigation, Project administration, Validation, Writing – original draft. GR: Data curation, Methodology, Project administration, Writing – original draft. SP: Formal Analysis, Funding acquisition, Project administration, Resources, Validation, Visualization, Writing – original draft. CY: Data curation, Funding acquisition, Resources, Supervision, Visualization, Writing – original draft, Writing – review & editing. ZL: Formal Analysis, Funding acquisition, Methodology, Project administration, Resources, Supervision, Validation, Visualization, Writing – original draft, Writing – review & editing.
